# Systematic Conservation Planning for Groundwater Ecosystems Using Phylogenetic Diversity

**DOI:** 10.1371/journal.pone.0115132

**Published:** 2014-12-16

**Authors:** Maria G. Asmyhr, Simon Linke, Grant Hose, David A. Nipperess

**Affiliations:** 1 Department of Biological Sciences, Macquarie University, Sydney, New South Wales, Australia; 2 Australian Rivers Institute, Griffith University, Brisbane, Queensland, Australia; University of York, United Kingdom

## Abstract

Aquifer ecosystems provide a range of important services including clean drinking water. These ecosystems, which are largely inaccessible to humans, comprise a distinct invertebrate fauna (stygofauna), which is characterized by narrow distributions, high levels of endemism and cryptic species. Although being under enormous anthropogenic pressure, aquifers have rarely been included in conservation planning because of the general lack of knowledge of species diversity and distribution. Here we use molecular sequence data and phylogenetic diversity as surrogates for stygofauna diversity in aquifers of New South Wales, Australia. We demonstrate how to incorporate these data as conservation features in the systematic conservation planning software Marxan. We designated each branch of the phylogenetic tree as a conservation feature, with the branch length as a surrogate for the number of distinct characters represented by each branch. Two molecular markers (nuclear *18S* ribosomal DNA and mitochondrial cytochrome oxidase subunit I) were used to evaluate how marker variability and the resulting tree topology affected the site-selection process. We found that the sites containing the deepest phylogenetic branches were deemed the most irreplaceable by Marxan. By integrating phylogenetic data, we provide a method for including taxonomically undescribed groundwater fauna in systematic conservation planning.

## Introduction

Aquifers store 95% of the world's available freshwater, and the groundwater within is the primary source of drinking water to an estimated 2 billion people worldwide [1]. However, groundwater is not only essential for human populations, but it is also vital to sustaining a wide range of groundwater dependent ecosystems such as rivers, riparian zones and estuaries [2].

The ultimate groundwater dependent ecosystems are those contained within the aquifer itself [3]. These environments harbor a rich diversity of organisms, and provide a variety of ecosystem services including bioremediation (the degradation of contaminants, including nitrates, by living organisms) and nutrient cycling [4].

As a substantial part of the ecosystem, groundwater invertebrates (stygofauna) contribute towards maintaining a healthy aquifer environment. This diverse fauna often has convergent morphology and cryptic species are common [5], [6]. Moreover, poor dispersal abilities have resulted in narrow distributions and high levels of endemism among stygofauna [7], making them particularly vulnerable to extinction in the face of increasing anthropogenic pressures [8].

Despite the apparent benefits of groundwater fauna in providing essential ecosystem services, most environmental policies are directed towards the protection of groundwater as a resource, not considering biodiversity [9]. Limited data on biodiversity and biology of stygofauna is one of the main reasons for the absence of appropriate conservation measures [10]. Globally, data on groundwater biodiversity and their distributions remain very patchy, although the fauna of some regions (e.g., southern Europe and North America) have been more thoroughly documented [11], [12]. In Australia, sampling efforts over the last two decades have revealed a great diversity of stygofauna, indicating that subterranean fauna potentially represent a significant amount of the continent's biodiversity [13] and therefore need to be protected in the same way as other freshwater systems [14].

The development of biodiversity protection schemes is increasingly being assisted by systematic conservation planning (SCP) frameworks [15], [16], [17]. SCP implements complementarity-based algorithms to systematically select sites with conservation features that are not represented by existing sites or reserves in order to represent maximum biodiversity over a minimum number of sites [18]. Conservation features have traditionally been species, ecosystems or surrogates thereof [19], [20]. However, recently the use of genetic data (microsatellite alleles) has been demonstrated [21].

SCP is increasingly utilized for freshwater conservation planning [14], [22] for rivers and wetlands, however, only a few studies have designed reserve networks for protecting stygofauna (France [8]; southern Europe [9]). In Australia, where the majority of groundwater fauna remain taxonomically undescribed [13] there is a need for innovative approaches for conservation of stygofauna, especially because rare or poorly sampled species strongly bias SCP analyses [17].

DNA barcoding (using cytochrome oxidase 1, *COI*, [23]) provides a surrogate method for identifying units of biodiversity and is particularly useful for rapid assessment of small, hyperdiverse or as yet undescribed fauna (e.g., ants [24]). However, it is difficult to delineate species with DNA since the appropriate level of sequence divergence for separating two species is unclear because this threshold varies between species and taxa [25].

Phylogenetic Diversity (PD) is a method for estimating biodiversity that side steps the need for identifying species and takes into account the evolutionary distinctiveness of organisms [26], [27]. The PD of a set of organisms is calculated by summing the branch lengths of the phylogenetic tree connecting that subset [26]. Because the tips on the tree can be species, populations, individual organisms or DNA sequences, PD is suitable for estimating biodiversity in poorly known ecosystems. Because molecular sequence data can be used to infer the phylogenetic relationship between a set of organisms, the PD approach offers a way of taking advantage of the wealth of information resulting from DNA barcoding programs [27], [28].

Several studies have dealt with the integration of PD into site selection algorithms [28], [29], [30], [31]. However, these algorithms are not implemented in the conservation planning software packages most frequently used by conservation practitioners. The Marxan conservation planning software package [32] is widely used for assisting decision makers regarding the placement of conservation areas (see [16]). More importantly, Marxan has become increasingly popular for use in freshwater conservation planning (mainly rivers) in Australia [22], [33]. Marxan uses a simulated annealing algorithm to minimize costs, while maximizing conservation features. It allows for the inclusion of a range of factors relevant for real-life conservation scenarios such as cost, habitat connectivity, and other socioeconomic factors [32].

The aim of this study is to explore the use of molecular sequence data and PD as surrogates for stygofauna diversity in systematic conservation planning using Marxan. We use taxonomically undescribed stygofauna in aquifers of the Hunter and Central coast regions of New South Wales, Australia, as a model system to demonstrate how phylogenetic branches can be used as conservation features in Marxan. This provides a framework to include data on largely unknown groundwater ecosystems into conservation planning, while still maintaining Marxan's ability to include costs and landscape connectivity. We amplified two different molecular markers (nuclear *18S* and mitochondrial cytochrome oxidase I) from groundwater crustaceans to evaluate how marker variability would impact on the site selection process. We compared the results of Marxan to that of the summed PD heuristic (as an upper bound for maximizing PD over the same number of sites) and rarefaction (lower bound) in order to evaluate the performance of Marxan in maximizing PD.

## Materials and Methods

### Sampling

**Figure 1:**
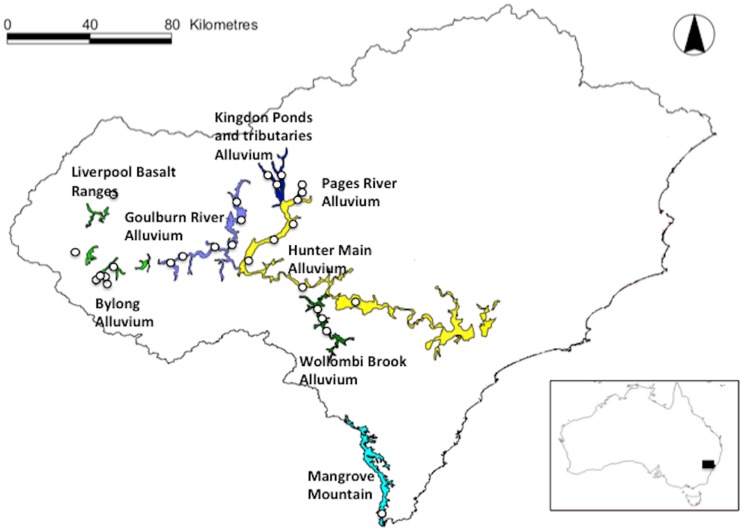
Map of the study region. Circles indicate location of groundwater bores containing stygofauna used in this study. Colours represent distinct groundwater systems as recognised by New South Wales Water. Inset map shows location of study region in Australia.

We collected groundwater samples from 26 groundwater-monitoring bores in the Hunter Valley region (Hunter alluvium, Kingdon Ponds and Tributaries alluvium, Wollombi Brook alluvium, Pages river alluvium, Liverpool Ranges Basalt) and Central Coast (Kulnura-Peats-Ridge-Mangrove Mountain plateau, Mangrove Creek borefield) of New South Wales (NSW), Australia (Fig. 1 and S1 Table), between Dec. 2010 and May 2012.

Sampling was restricted to existing bores that were owned and maintained by state (NSW Office of Water) and local government (Gosford Council) agencies for groundwater level monitoring. Access agreements were obtained from these government authorities. No additional permits or approvals were needed to sample the invertebrates because we were not targeting species listed as threatened under state or federal legislation, we were not sampling in designated protected areas and animal research ethics legislation for the state of New South Wales does not include invertebrates.

We collected water samples following the procedure outlined in Hancock and Boulton [34]. Water samples were taken from each bore using plankton nets or a motorized inertia pump. When pumping, 150–300 L of water was removed from the bore and subsequently passed through a 63-µm mesh sieve. Bores deeper than 30 m were sampled using a plankton net. The plankton net approach comprised hauling the net through the water column in the boreholes multiple times. Three hauls were made with a fine (63-µm) mesh net followed by three hauls with a coarser (100-µm) mesh net. The resulting water/sediment samples were combined and passed though a 63-µm sieve. The sample retained in the sieve was preserved in 96–100% ethanol and stored at −20°C. Samples were sorted under a light microscope and grouped as similar looking morphotypes, distinguishing the major crustacean groups found in groundwater; amphipods, isopods, syncarids, ostracods and copepods. Some groups were found in very high numbers (e.g. copepods was sometimes represented by thousands of individuals) in several samples and for the purpose of this study we only took out a couple of individuals for further analysis. While a variety of other invertebrates can often be present in groundwater we have only included crustaceans in this study.

### Molecular methods

For the purpose of this analysis, we limited the genetic analysis to crustaceans only. We extracted DNA from legs or from whole crustaceans (<2 mm in size) using the Bioline Genomic DNA extraction kit (Bioline, Australia) following the manufacturer's protocol for tissue samples. Where several representatives of a morphotype were found within the same bore, we extracted DNA from at least two individuals. We amplified short regions of the nuclear *18S* ribosomal DNA (rDNA) and the mitochondrial cytochrome oxidase I (*COI*) markers using the methods outlined by Asmyhr and Cooper [35] for *COI*. For *18S*, universal primers *18S*-1560-F and *18S*-2035-R were used under the following cycling conditions: Initial denaturation for 3 min at 94°C followed by 30 cycles of (94°C sec, 55°C for 30 sec, 72°C for 1 min) and a final extension at 72°C for 10 min. Polymerase chain reactions (PCRs) for were carried out in 25-µL volumes containing 3 µL of DNA template, 1 µm of each primer, 2 mm MgCl_2_, 2 µg BSA, 3 µL 5× Go TaqFlexiBuffer (Promega), 800 µm of dNTPs, and 1 U Taq Polymerase (Promega). PCR product was purified with ExoSAP-IT according to the manufacturer's instructions (USB Corporation). PCR products were sequenced by Macrogen (www.Macrogen.com). Partial mitochondrial *COI* and *18S* sequences were separately imported into MEGA5 [36], manually edited and aligned.

### Estimating Phylogenetic Diversity

Separate gene trees were constructed for each marker (COI, 18S). We used FindModel (Los Alamos National Laboratory: www.hiv.lanl.gov/content/sequence/findmodel/findmodel.html), the web interface of Modeltest [37] to determine the best fit of molecular evolution to the data. This model (GTR + Gamma distribution) was then implemented using a Bayesian framework for tree building in BEAST software v1.7.3 [38]. BEAST was run with a Yule speciation model and 15000000 generations (sampling at every 5000). For each marker, trees generated from BEAST runs were combined into a single target tree using TreeAnnotater v1.7.3 [38] with the first 300 trees discarded as Burn in and using the Maximum Clade Credibility (MCC) option.

Phylogenetic Diversity (PD) was calculated for each tree as the sum of the branch lengths connecting the individuals found at a particular bore [26]. These calculations always included the path to the root of the tree, including the basal branches connecting all taxa [30].

### Site selection procedure based on PD

**Figure 2:**
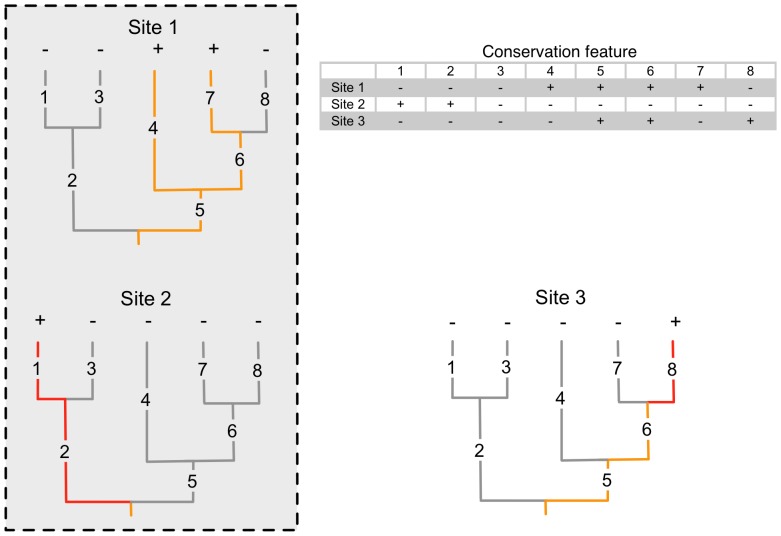
Interpretation of phylogenetic tree branches as features and its application to conservation planning. Tips of a phylogenetic tree are marked with + (presence) or - (absence) to indicate their representation for each of three sites. The subtree (in colour) that connects this set of tips to the root consists of a set of branches (numbered 1–8) that, when summed, constitutes the phylogenetic diversity of that site. Each of these branches are coded in the table as conservation features that are either present or absent for each site based on the presence or absence of the relevant tips. Branches marked in orange are represented in Site 1 while red branches are not represented in Site 1. Site 2 is the most complementary to Site 1 as it adds the most new features (and branch length). Therefore, in a scenario where Site 1 is already protected and only one other site can be chosen for protection, Site 2 should be added to the protected set (indicated by the grey box).

In the following analyses, we are treating phylogenetic tree branches as conservation features (see Fig. 2). It is important to note that in this case, the term “branch” refers to the edge length connecting nodes in the phylogenetic tree. As such, all branches are conservation features, not just the terminal nodes (leaves). Thus, each branch is coded as being either present or absent at each site (Fig. 2) as if they were separate species (or equivalent).

Furthermore, we consider each branch to be a surrogate for a set of features (i.e., characters), rather than a single feature, possessed by the organisms for which that branch is part of their lineage [26]. The length of the branch is indicative of the relative number of features, with longer branches representing far more feature diversity. The feature diversity of any location (hereafter referred to as a planning unit, PU) is therefore the total length of the set of branches representing the set of organisms present in that PU (see Fig. 2).

We used Marxan [32] to select PUs to include in a reserve network. Marxan aims to solve the ‘minimum set problem’, where the goal is to achieve some minimum representation of biodiversity features for the smallest possible cost [39]. In the following analyses we designated each groundwater bore as a PU.

In its simplest form, Marxan's objective function combines the total cost of the reserve system and a penalty for any of the ecological targets that are not met (Score  =  Cost of the reserve system + Boundary length of the reserve system + Penalty incurred for unmet targets). Because we used sampling points (groundwater bores) as planning units, the boundary length was not a component of our analysis. To put emphasis on longer branches (i.e., representing a large gain in PD) in the site selection process, we included a weighting system based on branch lengths. The length of each phylogenetic tree branch was divided by the depth of the tree, such that the total path length from leaf to root would be equal to one. These relative branch lengths were included as species penalty factors (SPF - a weighting factor that will be added to the objective function if the target for a conservation feature is not met in the current reserve scenario).

We set the cost of each PU to 1. We then ran the simulated annealing algorithm using different overall cost restrictions, from 2 (i.e, allowing the selection of two PUs only) to 10 (10 PUs). Marxan produces a selection frequency that measures how many times out of the total number of iterations for each run (we used 500) each planning unit was selected a part of the optimal set of sites. This provides an indication of how useful a PU is for creating an efficient reserve system [40], and is analogous to irreplaceability [41].

As a baseline to compare the performance of Marxan at maximizing PD over a set number of sites, we used a summed PD heuristic. This method is based on an algorithm described by Rebelo and Siegfried [42] as a means of using complementarity to prioritize areas for conservation. The algorithm proceeds by first selecting the area with the highest PD. The second area to be chosen will be that most complementary to the first – that is, the area resulting in the largest gain in PD when added to the first (Fig. 2). The algorithm then proceeds by choosing additional areas based on their complementarity to the set of those already chosen until the total PD is reached. At each step, ties (equal scores) are resolved by choosing at random from the available options. Because ties are resolved randomly, we repeated the algorithm 100 times and calculated the average ranking of each site. Calculations were done in R version 2.15.2 (R Core Team 2012).

We also calculated the corresponding rarefaction curve as a null model against which to compare the results of Marxan. The rarefaction curve shows the expected PD for a given number of planning units, selected at random and without replacement. The expected value of PD was calculated from the probability of each branch occurring in a set of planning units of a given size [43]. Calculations were done in R.

## Results

We obtained 75 *COI* sequences, however some of the sequences were in poor condition (19) and were excluded from further analysis. The remaining 56 included in the alignment represented 32 individual haplotypes. Sequences were between 200–800 bp, but because different taxa were amplified using different sets of primers, the final alignment, including all morphotypes, was substantially reduced (194 bp). We obtained 122 sequences of a short fragment of *18S* rDNA, of which 94 could be reliably interpreted and 22 were unique. There was considerable size variation in fragment size among taxa (150–350 bp). The final alignment had to be substantially shortened and resulted in an alignment of 70 bp, comprising a highly conserved region of this marker. Enough variation was present to separate between morphotypes. Sequences >200 bp were submitted to GenBank (accession numbers: KF361325- KF361330, KF361332- 361354, JX948792 – 948818).

The target (MCC) trees used for calculation of PD are included as (S1 Figure and S2 Figure). When comparing the results of Marxan based on the two different molecular markers, we found that for the analysis based on *18S* data with cost limitation to include two PUs only, 60% of total PD was represented in the two most irreplaceable PUs. When cost restriction allowed the inclusion of 10 PUs, the majority of PD in the dataset (90%) was represented among those 10 PUs. In contrast, for the same analyses based on *COI* data, two PUs represented only about 40% of total PD. When allowing the inclusion of 10 PUs, approximately 80% of PD was represented.

As such, the proportional gain in PD per additional PU added was greater for *COI* than for *18S*, but as expected (given the difference in variability of the two molecular markers), the total PD represented by 10 PUs was less for *COI* than for *18S*.

**Figure 3:**
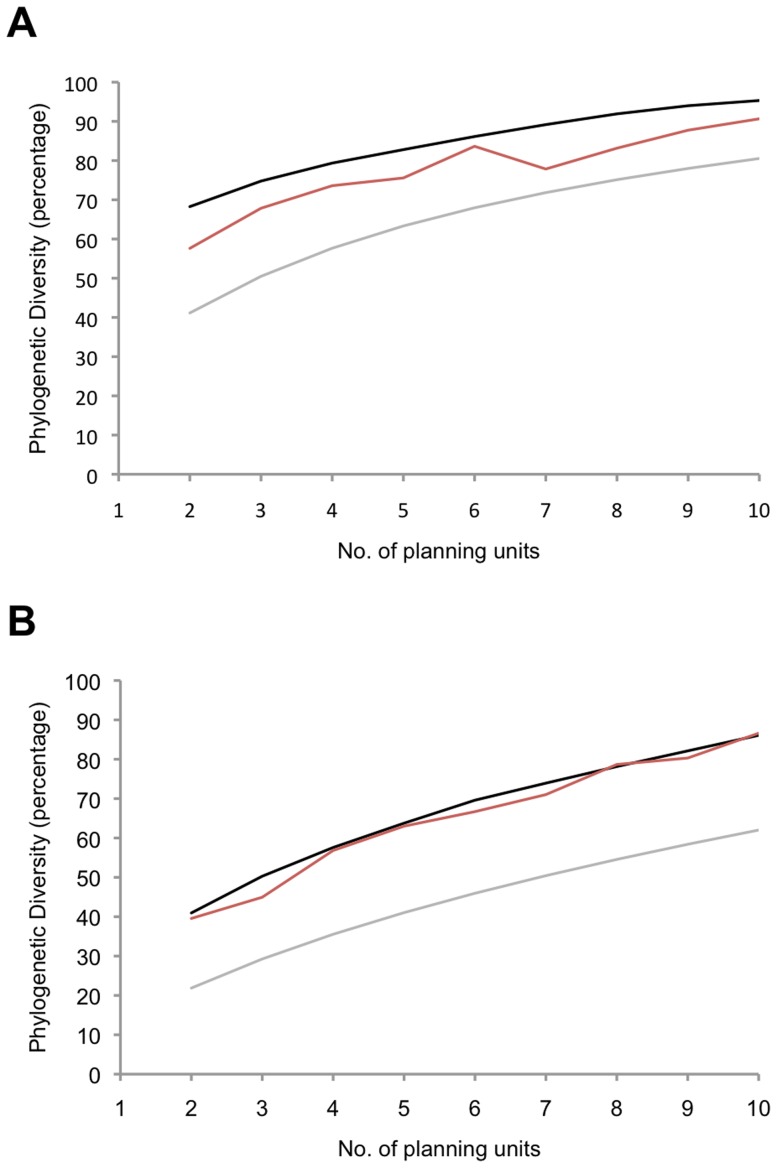
PD conserved by site selection for *18S* (A) and *COI* (B) for reserve selection algorithms. Black  =  Summed PD heuristic, Red  =  MARXAN, Grey  =  Rarefaction.

When comparing the result of Marxan to that of the summed PD heuristic, we made the following observations: 1) for the *18S* data, we observed a small difference between the amount of PD represented by the most frequently selected PUs by Marxan compared to the summed PD heuristics solution (Fig. 3a); and 2) for *COI*, the two algorithms performed equally in representing PD (Fig. 3b).

## Discussion

Here we use data from taxonomically undescribed stygofauna from New South Wales, Australia, as our model system to demonstrate how to include phylogenetic data into the systematic conservation planning using the software Marxan. While algorithms for maximizing PD over a minimum number of sites have been demonstrated in previous studies [29], [30], [31], the integration of PD into Marxan - or other similar operational biodiversity management frameworks (e.g., Zonation [44]) offers several advantages. These are: 1) in addition to maximizing biodiversity, these software packages can also take into account a range of additional variables, including habitat connectivity, conservation targets and socio-economic factors; and 2) they are constantly being developed and improved, including several freely available interfaces (e.g., C-plan, Zonae Cogito for Marxan) that can assist the analysis. Finally, Marxan is increasingly being applied to guide conservation plans for other freshwater habitats [14], thus our approach opens up the possibility of including groundwater biodiversity into a broader freshwater conservation plan, before detailed taxonomic or environmental data are available.

We do acknowledge that the Marxan solutions did not always maximise PD to the extent of the summed heuristic (Fig. 3), and this difference does require some consideration. Firstly, it must be realised that the summed heuristic is essentially a single solution. The most diverse PU is always chosen first and then proceeds from there in a particular sequence, except in the case of ties. In contrast, Marxan has (at least potentially) many solutions that are approximately equally optimal, and thus it is almost inevitable that the mean PD of multiple Marxan solutions will be less than a single summed heuristic solution. Secondly, in the absence of differences in cost or boundary length between PUs, Marxan solutions become dependent on the penalty applied when certain features (branches) were not selected. We made this penalty proportional to branch length, summing to one for any particular path from root to tip. What is not presently known is how best to apply this penalty to achieve the goal of maximising PD. A detailed simulation analysis is required to fully test the implications of different penalty weightings, as well a different tree topologies, on Marxan solutions. Despite the differences between our methods, we still assert that conservation planning algorithms, such as Marxan, should be preferred over simple optimisations such as our summed heuristic, both for their flexibility and for their capacity to take factors such as differential costs or boundary lengths (connectivity) into account, although work still needs to be done on optimising these procedures for conserving PD.

Representing the maximum number of conservation features over a minimum number of sites is a general goal in systematic conservation planning [18]. Aiming to include all species, or in our case, branches of a phylogenetic tree, would probably be applicable for the majority of ecosystems, because most species are generally found in more than one site within a restricted geographical region. For stygofauna, where the majority of species seem to have extremely narrow distributions and high levels of endemism [7], [9], conserving all species would often mean that the majority of PUs would have to be included in the final reserve network, a highly unrealistic conservation goal. Using PD as a measure of biodiversity in groundwater offers an advantage over, for example species richness, because it allows an estimate of the phylogenetic distinctiveness of a particular taxon (or haplotype) and how much it contributes to overall diversity.

Michel et al. [9] noted that incomplete sampling combined with narrow distributions of most stygofauna resulted in little flexibility for the placement of a groundwater reserve network when based on species data. In such a situation, the additional information resulting from the use of molecular data and PD could provide more flexibility of which PUs to include or exclude. Faith and Baker [28] illustrate this point using an example with freshwater crayfish. Molecular data revealed that two closely related sister species were found in two different locations. When anthropogenic actions caused the extinction of one of them, the location containing the remaining sister species was assigned a greater conservation priority based on PD, because it now solely represented the deeper branch (and thus features) shared by the two sister species [28].

When using phylogenetic branches as conservation features, the site selection process in Marxan is driven by the tree topology. Internal branches, which may be long and thus represent a relatively large amount of PD, often split into several terminal nodes that may all be found in different PUs (see Fig. 2). Because of this, relevant internal branches are always represented within the prioritized PUs. However, a deep branch can sometimes also represent one distinct lineage only present in one PU. It is therefore less likely to be represented among the prioritized PUs. To ensure that such branches were selected, all branches were weighted by their relative length (and thus contribution to PD) and this weight was included as a species penalty factor in Marxan. Marxan ranked the sites containing the longest branches highest (i.e., they were deemed most irreplaceable). When the sites with the deepest branches are included in the reserve network, the remaining sites will only contribute a marginal gain in PD (and thus feature diversity). Hence, conservation planners may choose to exclude some of those marginal gain PUs (e.g., containing taxa that are already represented by a close relative), resulting in more flexibility.

The number of sites that are needed to include the majority of the deep branches will depend on the resolution of the genetic marker used for estimating phylogenetic relationships. The *18S* marker is most frequently used to detect higher taxa (e.g., class, family, order level) [45], whereas *COI* is extremely variable and can be used to delineate between closely related species or populations [23], resulting in different tree topologies. The *18S* tree had higher taxa (e.g., Amphipoda) within the tree forming compact groups with long unbranched stems leading up to them (i.e., stemmy tree), whereas the *COI* tree had relatively longer inter-nodal distances towards the tips of the phylogeny (i.e., tippy tree) [46]. The two most irreplaceable PUs based on the *18S* marker represented almost 60% of the total PD (Fig. 3a). The remaining PUs contained short branches of similar size (i.e., within taxa variation) so subsequent choices of PU made less impact on PD (Fig. 3a). As a result, the selection procedure was less constrained in the way additional sites were chosen, and we observed a slight difference in the ranking of sites between Marxan and the optimal curve of summed PD heuristics (Fig. 3a). However, the difference in PD among the final solutions (10 PUs) is marginal, and most of the total PD (>85%) is represented, suggesting that most higher taxa are represented in the final reserve solution. In contrast, for *COI* the first two PUs represent only 40% of total PD, and each subsequent PU selected adds a significant amount of PD to the reserve network (Fig. 3b).

A drawback with using *COI* is that saturation of nucleotide substitutions can result in homoplasy, and may affect the accuracy of estimates of species divergence and relationship [47]. Being more useful for solving deeper phylogenies, *18S* may better predict evolutionary relationships [45], and thus possibly functional diversity [48]. For groundwater ecosystems, which carry out a range of valuable ecosystem services [4], representing and conserving functional diversity is of major importance. However, cryptic diversity is common for stygofauna and is generally resolved using *COI* [5], [6]. Moreover, using *COI* would promote more flexibility in the site selection procedure because it allows delineation of closely related species, and also may include significant diversity not found in *18S*.

While it is true that absence of data on groundwater fauna hinders efficient conservation, this is not a problem unique to groundwater ecosystems. In freshwater conservation planning both biotic and abiotic indicators are used as surrogates for biodiversity [14], for example by using coarsely defined biological attributes including zoogeographic zones or freshwater ecoregions [49]. However, for coarse filter surrogates to be efficient in representing wanted taxonomic groups, they should be based on data on how species and communities respond to the physical and chemical environment [14]. Thus such surrogates should be applied with caution and preferably be tested for efficiency [50]. For stygofauna, a few attempts have been made towards developing predictive models for distribution (e.g., GW index [51]; Indicator species [52]) but these approaches have yet to be tested and standardized across different regions. There is a strong impetus to fully and systematically integrate groundwater ecosystems into conservation plans since subterranean ecosystems are increasingly being considered as part of environmental impact assessments in the face of increasing mineral, oil and gas development [53].

In this study we attempted to demonstrate how molecular data and PD can be combined as surrogates for stygofauna diversity in systematic conservation planning using Marxan. However, our study has several limitations that need to be addressed before this is applicable to a real conservation planning scenario. Firstly, we designated the groundwater monitoring bores as planning units, even though some of them accessed the same aquifer. Future studies could analyze clusters of bores accessing the same aquifer as the same PU, thus designating each aquifer as a planning unit. This would facilitate the assignment of appropriate threats and socio-economic factors for each PU [8], and these should be integrated as costs in the objective function prior to the analysis. Furthermore, if including groundwater into a broader freshwater conservation plan, the connectivity between catchments and groundwater (i.e., measured by the rate of recharge) can only be assessed at the aquifer scale. Moreover, while we focused on the crustacean stygofauna only, future studies could easily reduce cost and effort, as well as increase the number of taxa included in the analysis by using a meta-barcoding approach in which total DNA can be extracted and amplified from environmental samples [54]. Finally, we would like to emphasize that this is not an attempt to discourage standard taxonomy and the use of species data in conservation. However, for ecosystems such as groundwater ecosystems, where development and disturbance from expanding mining activities may eradicate the fauna before they are even discovered [10], our method may help to buy some time to allow the discovery and description of some unique and valuable ecosystems and bioresources.

## Supporting Information

S1 Figure **Maximum Clade Credibility (MCC) target tree based on** ***18S*** **data.** Tips are labeled and coloured according to bore ID number.
(TIFF)

S2 Figure **Maximum Clade Credibility (MCC) target tree based on** ***COI*** **data.** Tips are labeled and coloured according to bore ID number.
(TIFF)

S1 Table **Bore ID and corresponding latitude/longitude coordinates for each groundwater bore.**
(DOCX)
