# Supplementary figures and images for: Systematic Conservation Planning for Groundwater Ecosystems Using Phylogenetic Diversity
Source: PLoS One. 2014 Dec 16;9(12):e115132. doi: 10.1371/journal.pone.0115132 (PMC4267811; doi:10.1371/journal.pone.0115132)

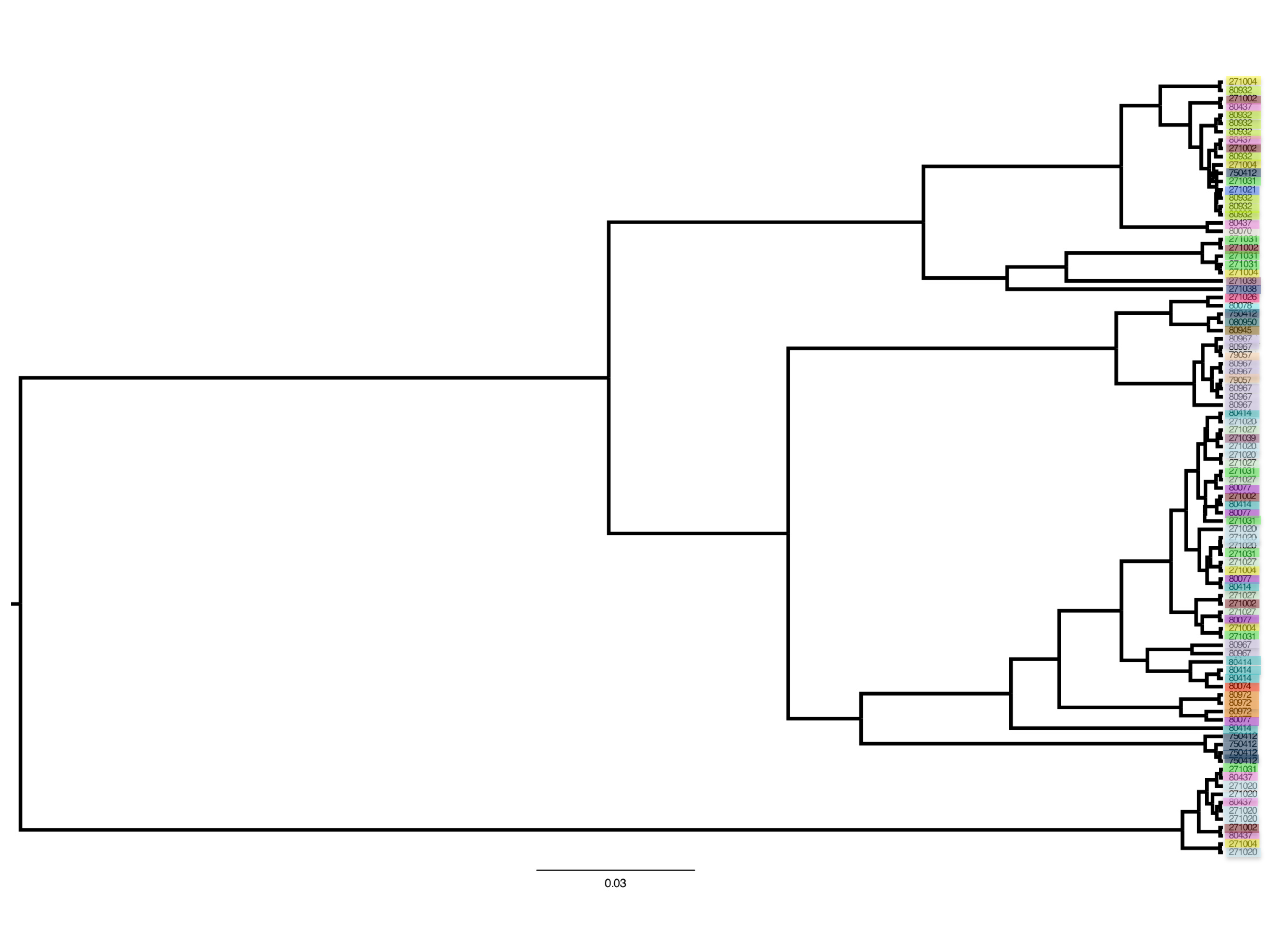

Supplement: S1 Figure — Maximum Clade Credibility (MCC) target tree based on 18S data. Tips are labeled and coloured according to bore ID number. (TIFF) [file pone.0115132.s001.tiff]

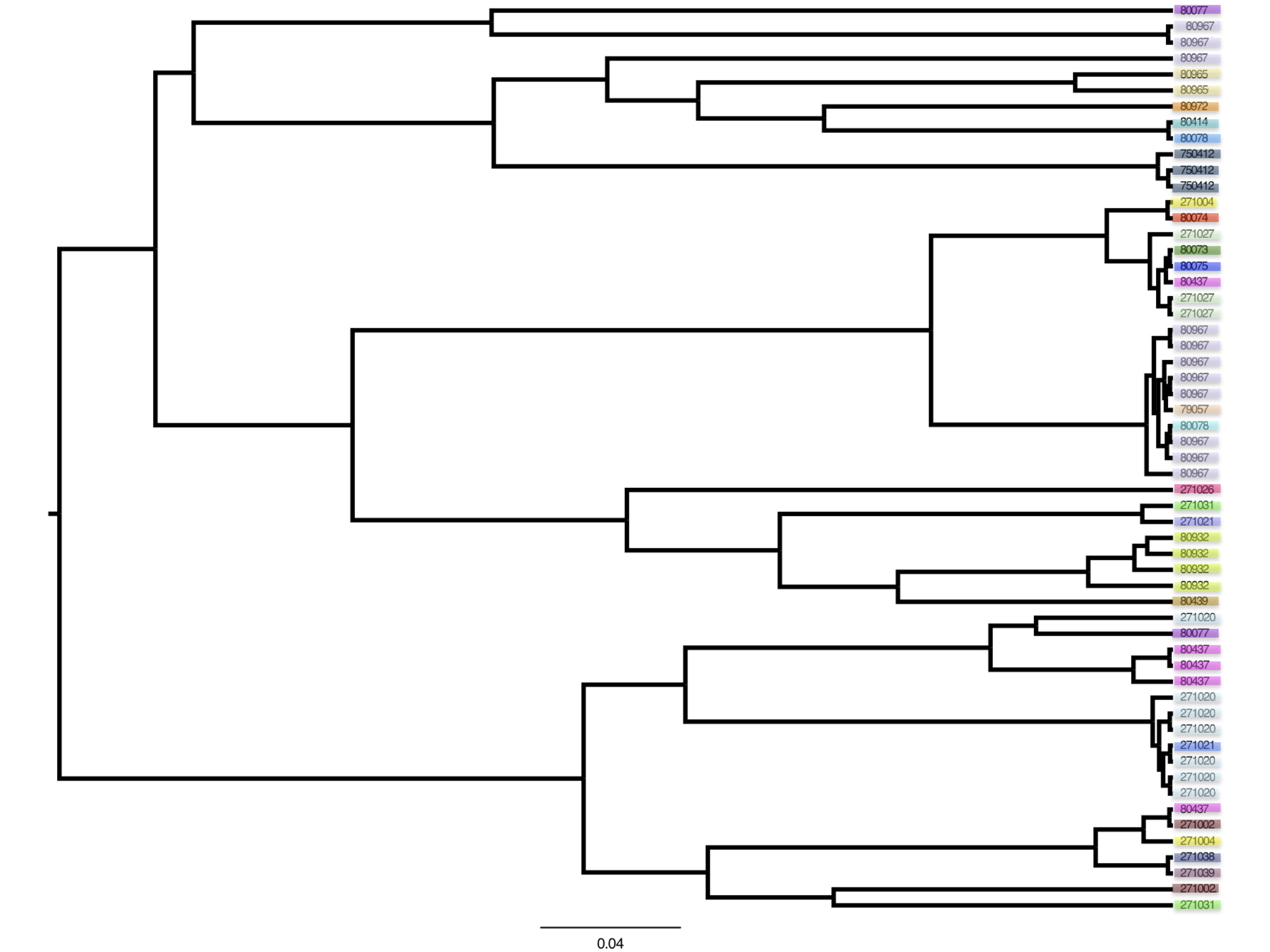

Supplement: S2 Figure — Maximum Clade Credibility (MCC) target tree based on COI data. Tips are labeled and coloured according to bore ID number. (TIFF) [file pone.0115132.s002.tiff]
