# Supplementary material for: Systematic Conservation Planning for Groundwater Ecosystems Using Phylogenetic Diversity
Source: PLoS One. 2014 Dec 16;9(12):e115132. doi: 10.1371/journal.pone.0115132 (PMC4267811; doi:10.1371/journal.pone.0115132)
Supplement: S1 Table — Bore ID and corresponding latitude/longitude coordinates for each groundwater bore. (DOCX) [file pone.0115132.s003.docx]

| Bore ID | Latitude | Longitude |
| --- | --- | --- |
| 79057 | -32.7072328 | 151.0519236 |
| 80070 | -32.03980142 | 150.8609397 |
| 80073 | -32.03624067 | 150.855306 |
| 80074 | -32.09067082 | 150.8608035 |
| 80075 | -32.39599681 | 150.6920138 |
| 80077 | -32.38958582 | 150.7103364 |
| 80078 | -32.39554953 | 150.6885182 |
| 80414 | -32.53355014 | 149.9939686 |
| 80437 | -32.04664931 | 150.8205316 |
| 80439 | -32.0475673 | 150.9441991 |
| 80932 | -32.09534361 | 150.1337796 |
| 80945 | -32.2542252 | 150.6301899 |
| 80950 | -32.38492429 | 150.464648 |
| 80965 | -32.69554567 | 151.0070772 |
| 80967 | -32.75114274 | 151.1027977 |
| 80972 | -32.30244337 | 150.9334976 |
| 271002 | -32.37966351 | 150.1311476 |
| 271004 | -32.44723623 | 150.0719047 |
| 271020 | -31.9857675 | 150.8149251 |
| 271021 | -32.0933289 | 150.9409962 |
| 271026 | -32.5615426 | 151.1224249 |
| 271027 | -32.0721606 | 150.9448605 |
| 271031 | -32.4545264 | 150.8524576 |
| 271038 | -32.531944 | 150.121389 |
| 271039 | -32.508056 | 150.123333 |
| 750412 | -32.7072328 | 151.0519236 |

Table S1 Bore ID and corresponding latitude/longitude coordinates for each groundwater bore.
